# An ERP Analysis of Recognition and Categorization Decisions in a Prototype-Distortion Task

**DOI:** 10.1371/journal.pone.0010116

**Published:** 2010-04-12

**Authors:** Richard J. Tunney, Gordon Fernie, Duncan E. Astle

**Affiliations:** 1 School of Psychology, University of Nottingham, Nottingham, United Kingdom; 2 School of Psychology, University of Liverpool, Liverpool, United Kingdom; 3 Department of Experimental Psychology, University of Oxford, Oxford, United Kingdom; University College London, United Kingdom

## Abstract

**Background:**

Theories of categorization make different predictions about the underlying processes used to represent categories. Episodic theories suggest that categories are represented in memory by storing previously encountered exemplars in memory. Prototype theories suggest that categories are represented in the form of a prototype independently of memory. A number of studies that show dissociations between categorization and recognition are often cited as evidence for the prototype account. These dissociations have compared recognition judgements made to one set of items to categorization judgements to a different set of items making a clear interpretation difficult. Instead of using different stimuli for different tests this experiment compares the processes by which participants make decisions about category membership in a prototype-distortion task and with recognition decisions about the same set of stimuli by examining the Event Related Potentials (ERPs) associated with them.

**Method:**

Sixty-three participants were asked to make categorization or recognition decisions about stimuli that either formed an artificial category or that were category non-members. We examined the ERP components associated with both kinds of decision for pre-exposed and control participants.

**Conclusion:**

In contrast to studies using different items we observed no behavioural differences between the two kinds of decision; participants were equally able to distinguish category members from non-members, regardless of whether they were performing a recognition or categorisation judgement. Interestingly, this did not interact with prior-exposure. However, the ERP data demonstrated that the early visual evoked response that discriminated category members from non-members was modulated by which judgement participants performed and whether they had been pre-exposed to category members. We conclude from this that any differences between categorization and recognition reflect differences in the information that participants focus on in the stimuli to make the judgements at test, rather than any differences in encoding or process.

## Introduction

A fundamental aspect of human cognition is the ability to acquire knowledge of categories. This enables us to assign properties to an object that we have learned are common to other members of that category. This has clear survival value, for instance we may be reluctant to eat a plant with milky sap that we have not encountered before if we have learned that other plants with milky sap are poisonous. We should infer that the new plant is also likely to fall into the category of poisonous plants.

Precisely how the mind represents categories has received a substantial amount of attention and recently theories of categorization have been informed by studies involving amnesic patients and functional imaging. The present research is concerned with two classes of theory of categorization in particular: episodic models and prototype models. Both prototype and episodic models assume that categorization decisions are based on similarity. According to episodic models we memorize each instance of a category [1]. When asked to decide whether novel items are category members or not, the decision is based on a comparison of the item with each stored exemplar. In effect categorization is little more than a form of episodic memory. By contrast, prototype models assume that the categorization decision is based on the similarity of the item to a prototype, rather than to stored exemplars [2]. A prototype is usually defined as an abstraction of the central tendency or an average of previously encountered exemplars. The exemplars themselves need not be stored in memory giving prototype theory an economical advantage. Exemplar theory has the advantage of computational simplicity. There are a number of different candidate models within each class. We refer to episodic models as any model that describes categorization as essentially a memory based process as distinct from models that assume some form of abstraction occurs during learning as is described by prototype models. We discuss in more detail one episodic model that is based on exemplar similarity [3], although it is our intention to compare episodic models generally with prototype models rather than any one specific episodic model.

A method that is frequently used to decide between these two classes of model is to identify dissociations between categorization and recognition. That is, if different patterns of data are observed when experimental participants are asked to make recognition decisions for category members that they have previously encountered compared to when they are asked to make decisions about the category membership of novel items, the conclusion that is often made is that these two decisions recruit different processes [4], [5], [6]. The same logic is often used in studies that attempt to understand many cognitive processes that appear to involve separate processes, such as between implicit and explicit learning, re, priming and recognition, and recollection and familiarity based memory. Typically however, studies of this kind compare responses to different stimuli. In many respects it seems sensible to use stimuli in a recognition test that have previously been memorized and to compare this to a categorization test using novel stimuli. However, it is inevitably unclear whether any observed differences in behaviour are due to the differences in the stimuli (old items are by definition more familiar than novel items), rather than differences in the processes used to make the decisions. That is, when dissociations between categorization and recognition are based on different stimuli it is difficult to determine if the reported differences are due to the underlying processes, rather than some difference in the stimuli. A convincing dissociation would be apparent when it is observed in different decisions about the same stimuli. The principle aim of this paper is to compare recognition and categorization using the same set of stimuli. If differences in behaviour are observed in this case then we can conclude that these two kinds of decision do indeed recruit different processes. Because many previous studies have used neuropsychological methods to dissociate processes, and because similar behaviour can arise from different underlying processes, we examined both ERP activity and behavioural responses for recognition and categorization.

**Figure 1:**
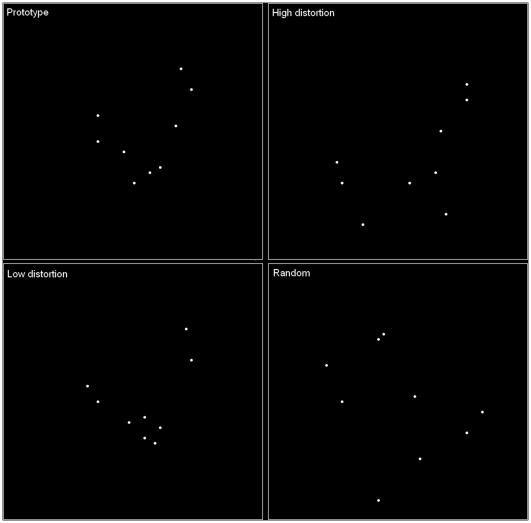
Example Stimuli.

Prototype-distortion tasks have been influential in developing our understanding of how knowledge of categories is acquired [7]. This particular paradigm is useful, because it permits the study of how participants learn information, whether by memory or abstraction, that is unlikely to be influenced by prior knowledge. In this paradigm a prototype stimulus is formed by generating a random pattern of nine dots, and additional category members are created by distorting the coordinates of each dot of the prototype (see Figure 1). In the standard preparation participants are first shown a set of category members but not the prototype stimulus itself. In a subsequent test participants are shown a set of previously unseen patterns that consists of category members that vary in their similarity to the prototype, along with the prototype item itself, and category non-members. Numerous studies report that participants are more likely to endorse items that are similar to the prototype, including the prototype, as category members than dissimilar items [8]. This pattern of results is often interpreted as evidence that participants abstract a representation of the category that closely matches the prototype even though this is not present in the study period. This contrasts with an alternative model that assumes categories are represented by storing previously encountered instances in episodic memory [9].

Evidence from studies of amnesic patients and brain imaging support the prototype abstraction model of categorization by showing dissociations between categorization and recognition of study items. These suggest that categorization is predicated on a set of neural processes different from memory of the study items. [4], [5], [10]. The rationale for this is that if the prototype is abstracted during the study episode then episodic memory would not be required to store the study exemplars. It follows that knowledge of the category could be acquired by patients with organic amnesia. Indeed, at least three studies have found similar patterns of categorization in amnesic patients and healthy controls [4], [5], [10]. However, the amnesic patients performed at chance in a subsequent recognition test of the study exemplars. By contrast the control participants performed much better in the recognition test. The conclusion from prototype-distortion studies in amnesic patients is that category knowledge can be acquired in the absence of memory for study exemplars.

Data from a number of *f*MRI studies also lend support to this model. These tend to be concerned with activity that occurs when participants are asked to make decisions at test, rather than activity that might result from prototype abstraction during the study episode. The first study of this kind reported decreased activity in regions of the posterior occipital cortex for category members relative to category non-members [11]. One possibility is that category members are processed more fluently than non-members. An increase in activity was observed in frontal cortical areas that may be related to conscious deliberation of whether an item is a category member or not [11]. A related study [12] replicated the finding that the posterior occipital cortex shows a decrease in activation for category members relative to category non-members. Moreover, a separate recognition task revealed increased activation in the frontal and temporal lobes and, importantly, that the posterior occipital cortex showed increased activation. This finding appears to show a dissociation in the kind of activation resulting from categorization and recognition decisions. A possible interpretation may be that categorization relies on processes akin to perceptual priming and perhaps familiarity based memory [11]. However, the activation of different neural regions may also be influenced by how the participants are instructed to learn the category. For example, different patterns of activity have been observed when participants categorize test items following either incidental or intentional learning instructions during the study episode [6]: intentional learning results in activation of the hippocampus; by contrast incidental learning results in deactivation of the posterior occipital cortex. Other studies indicate that explicit memory might be involved in the early stages of learning as shown by hippocampal activation, but this declines as knowledge of the category is acquired [13], [14].

Despite the evidence in favour of prototype abstraction an alternative episodic model proposes that categorization is based merely on exemplar similarity. According to this model [9], [15] participants make categorization judgements on the basis of the similarity of the test items to an episodic representation of the study items. Dissociations between categorization and recognition arise from a more liberal criterion for accepting test items as category members than for accepting test items as previously encountered. This model has had some success in accounting for behavioural data in healthy participants. How then can this model account for the preserved capacity to form categories in amnesic patients? The model does this by assuming that episodic memory is impaired but not entirely eliminated by organic amnesia [16]. In this way a liberal response criterion results in preserved categorization. It is not clear however, how this model can account for the data obtained from *f*MRI studies, which show qualitatively different patterns of activity for recognition and categorization, unless these effects result from the use of different stimuli in the categorization and recognition tests.

Event Related Potentials (ERP) can also provide potentially useful information about the neural correlates of category learning [17], but this technique has not previously been used in this specific paradigm. This method has the advantage over *f*MRI in that categorization and recognition can be disambiguated by differences in both timing and region. One previous experiment that used different materials (blobs rather than patterns of dots) found different ERPs for categorization and recognition [17]. Early visual potentials (N1, 156–200 msecs) were associated with category membership. The amplitude was significantly more negative for category members than for non-members. These data are consistent with *f*MRI studies that implicate the posterior occipital cortex in categorization [11], and support the view that this region (and categorization) is predicated on largely visual processes [18]. Middle latency components (FN400, 300–500 msecs) were associated with both category membership and with recognition. This component is interesting because it is thought to underlie familiarity based processing in dual-process theories of recognition memory [19], [20]. Later potentials in parietal regions (400-msecs) were associated with recognition only. This component is related to explicit recognition (i.e. recollection) of information from previous study episodes [20], and is thought to result from deeper hippocampal and medial temporal lobe activity because it is absent in patients with lesions of the hippocampus [21]. Because the timings of these effects are so brief differences between categorization and recognition are not likely to be detected using *f*MRI. If there are differences in the processes recruited by categorization and recognition we believe that they are most likely to be found using ERPs.

In the experiment that follows we compare the ERPs of categorization and recognition in the prototype-distortion task. The experiment is necessary because all of the previous neuropsychological investigations of this task have been conducted using *f*MRI, amnesic patients, or both. Moreover, given that prototype abstraction is typically related to early visual processing, ERPs, with their high-temporal resolution, ought to provide the ideal technique to study these processes [18]. The previously mentioned examination of ERPs in categorization was conducted using a different kind of stimuli [17] and it is necessary to determine if the same processes and neural mechanisms are involved in this task. The experiment is also important because it introduces a methodological advance over the previous ones. All of the studies mentioned previously have compared recognition and categorization tasks using different items, because the categorization tasks require previously unstudied category members and recognition tasks require that at least half of the test items have been studied before. Because these previous studies have used different items in their categorization and recognition tests it is conceivable that the reported dissociations have occurred because of differences in the stimuli rather than purely differences in the processes underlying the decisions. In the experiment that follows two groups of participants made either recognition or categorization decisions about the same set of test items. Crucially, for the recognition group the category members used in the test were also used during the study period as category exemplars. The categorization group differs in that a different set of category exemplars was used for the study items. In this way we can ensure that any differences between categorization and recognition are due to the underlying cognitive process and not to perceptual differences between the items used in the two tests. This control of stimulus equivalence is also crucial for the ERP comparison. There were also two control groups who made either recognition or categorization decisions about the same sets of items but who saw no exemplars in the study period. Two previous comparisons of categorization and recognition in prototype-distortion tasks found that in terms of behavioural accuracy recognition was superior to categorization [10], [22]. These studies did use different items for each test, but if as the authors claim there is a process difference between the two kinds of decision we should obtain similar results even when, as in the experiment that follows, the stimuli are identical. On the other hand, if the two decisions involve the same underlying process, and if the previous results are due to differences in the items, then there should be no difference in the accuracy of recognition and categorization behaviour. Predications about the precise ERP components that we might observe are necessarily speculative. However, generally the prototype model would predict different ERP components for categorization than for recognition. For instance Early visual potentials (N1, 156–200 msecs) [17] for categorization and latter parietal potentials (400-msecs) for recognition [20]. Models such as the exemplar or episodic accounts that claim that categorization and categorization are predicated on the same underlying processes are likely to predict similar components.

## Methods

### Ethics statement

This study was approved by the ethical review board at the School of Psychology, University of Nottingham, UK. Written consent was obtained from all the participants who were free to withdraw from the study at any time.

### Participants

Sixty-three right-handed volunteers took part in the experiment. Their mean age was 26 years (sd  = 5.35); 40 were male and 23 were female. The participants were paid £20 (approx. €27, US$40).

### Stimuli

The stimuli consisted of dot-patterns constructed using the method described by Posner, Goldsmith and Welton [7]. Using this method we first created a prototype pattern and then we created three lists each with 40 items. Distorting the coordinates of the prototype pattern created List 1 and List 3 items. List 2 items were pseudo-random patterns (see Figure 1).

### Design and Procedure

This was a 2×2×2 mixed model design with Exposure (Pre-exposed vs. Control) and Instructions (Recognition vs. Categorization) as between-subjects factors and List (1 vs. 2) as a within-subject factor. In each case these lists were composed of the same items.

The experiment consisted of a study period and a test period. During the study period the pre-exposed participants were presented with either List 1 items in the Recognition Condition, or List 3 items in the Categorization condition. The participants were told that the study was an experiment on visual attention and were asked to look for the dot closest to the centre of the screen but were not given any instructions about the presence of a category or how to encode the items. Study trials consisted of a 3000 msec white fixation cross, followed by a study item that appeared for 5000 msecs with a white frame. The control participants were informed that they were taking part in an experiment on subliminal perception and visual attention. Control ‘study’ trials consisted of a 3000 msec white fixation cross. After this a black screen was displayed for 1000 msec, then the screen flashed white for 50 msecs, followed by a black screen with an empty white box visible for 50 msecs, followed by another white screen for 50 msecs before an empty black screen returned for 4000 msecs. The control participants were also asked to try and identify the central dot in each pattern but that they would be presented very briefly and be difficult to see. After the end of the study period there was a short break during which the electrodes were checked. No EEG was recorded during the study period.

Prior to the test the participants in the Categorization conditions were told that all of the items that they had just seen were instances of a category and that they would now see some new items, some of which belonged to the category and some did not. Each test trial consisted of a 3000 msec fixation cross. Each test item appeared for 5000 msecs followed by a prompt to indicate whether the item was category member or not. The participants in the recognition conditions were told that they would be given a recognition test for the items that they had just studied.

We presented the same 40 category members and 40 category non-members to the four groups during the test phase. The two Pre-exposed groups had already been presented with category members during the study phase. For the Pre-exposed Recognition group these were the same 40 category members as were subsequently used in the testing phase (i.e. List 1). For the Pre-exposed Categorization group the study items were different category members to those we subsequently used for the test phase (i.e. List 3). By changing the study items in each condition, List 1 items were ‘old’ for the recognition group because they had also appeared as study item. For the Categorization group the List 1 items were new in the sense that they had not appeared in the study period but belonged to the same category as the study items.

### ERP recording and ERP formation

EEG was recorded throughout each block in the test-phase using a 128-channel electrical geodesic net (Electrical Geodesics, Inc.: EGI) [23], digitised at 250 Hz. The recording was performed with a hardware bandpass filter of 0.01 Hz to 100 Hz. Before recording, impedance on each of the 128 electrodes was reduced to <50 kΩ. Due to amplification techniques, the EGI system provides an excellent signal-to-noise ratio, despite these relatively high electrode impedances [24]. The vertex was used as an acquisition reference.

Stimulus-locked epochs were created, time-locked to each test item, with each epoch starting 100 ms before stimulus onset and ending 1000 ms afterwards. Segments were rejected if contaminated by eye-blinks/movements (indicated by EOG activity greater that 70 µV). This process was also checked manually. Trials containing voltage amplitudes greater than 200 µv or a change greater than 100 µv were also removed. We did not reject ‘error’ trials, as control participants who have never seen the items before cannot correctly ‘recognize’ them. Instead we objectively classified stimuli as to whether they were List 1 or List 2 items. ERPs elicited by these two types of stimuli were compared across the four groups of subjects. The average waveform for each stimulus type, for each subject, comprised at least 25 individual trials.

### Waveform comparisons

**Figure 2:**
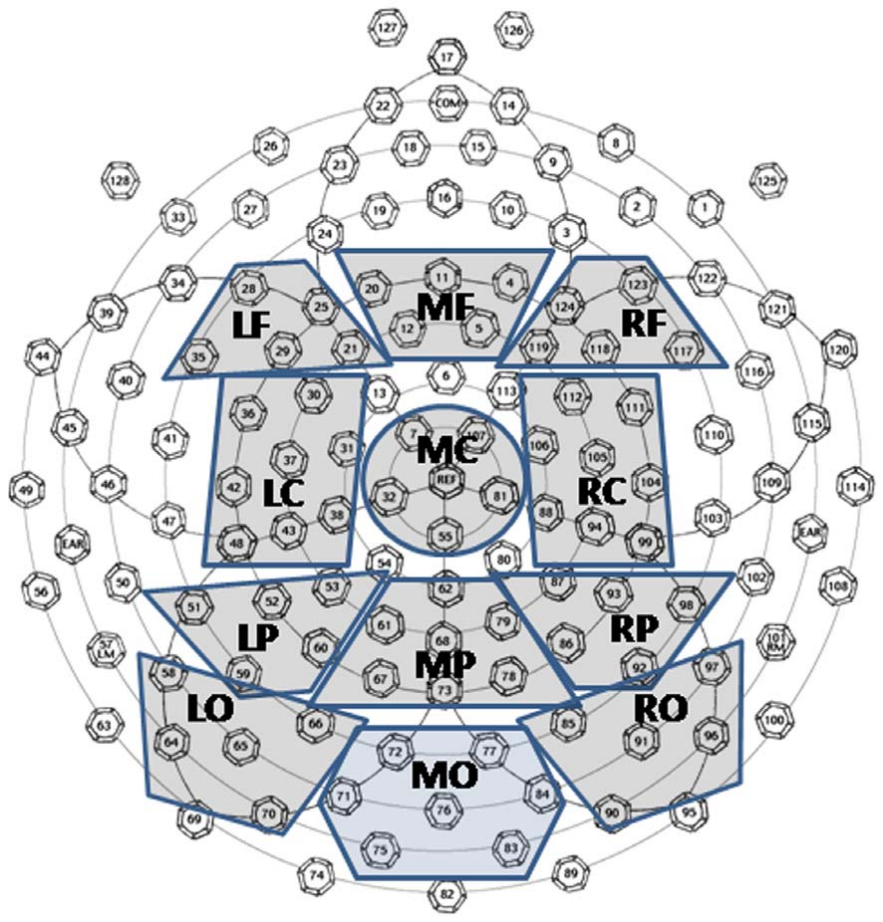
Electrode Montage.

Segments were average-referenced to a standard adult 128-electrode montage. Epochs were baseline-corrected for the first 100 ms before the onset of the stimulus. We formed clusters of electrodes, as means of data reduction. By using clusters rather than individual electrodes we were able to cover a large portion of the scalp and still include ‘electrode position’ in our ANOVAs, alongside the other within- and between-subjects factors, without the ANOVAs become uninterpretable. Our clusters covered 71 electrode sites, and were organised into 12 clusters, one around each of the following electrodes: F3, Fz, F4, C3, Cz, C4, P3, Pz, P4, PO3, Oz and PO4. The specific clusters that we used can be seen in Figure 2.

We created four time-bins, based upon the preceding literature, and compared the mean amplitude values across each bin. These are labelled as the early bin (160–200 ms, post item onset), the mid-latency bin (320–480 ms) and the late-latency bin (480–800 ms). In each bin we used a mixed-design ANOVA. This always comprised the between-subjects factors of Exposure (two levels, Pre-exposed versus un-exposed Control groups), and Instructions (two levels, Recognition versus Categorisation groups). The ANOVA also always included the within-subjects factor of List (two levels, List 1 vs. List 2). This enabled us to test whether List 1 and List 2 items elicited different ERPs, and whether the evoked response to the items was influenced by the participants' prior experience and judgement type. It also enabled us to test whether or not these factors interacted with one another.

In addition to these three experimental factors we included electrode cluster location in our ANOVAs, to test whether the distribution of any of the above effects differed across the conditions. Electrode cluster location was entered as two factors: cluster position along the left-to-right lateral axis (three levels, subsequently labelled electrode [L-R]) and cluster position along the anteroposterior axis (four levels, subsequently labelled electrode [A-P]).

All of our analyses were initially conducted on unscaled data. However, where this revealed an interaction between any of the experimental factors and any of the electrode factors we recalculated the ANOVA using data scaled according to McCarthy and Wood's [25] rescaling technique. The logic behind this was as follows: with un-scaled data the interaction between the experimental and electrode factors is necessarily ambiguous; an interaction could arise from a genuine difference in the distribution of the effects across the two conditions, or simply from the main effect having a multiplicative effect across the electrodes. Scaling results in data normalisation; with the main effect removed, one can then test for a genuine interaction between that experimental factor and electrode position. It is, however, worth noting that this technique is not perfect: Urbach and Kutas [26] demonstrated that this approach can fail to properly take account of the main effect, resulting in incorrectly reporting a significant interaction with electrode location; in some cases this normalisation may produce the opposite effect, masking genuine topographical differences. However, this is the most recognised means of disambiguating interactions involving electrode location, and as such we applied it to our data where necessary. That said, any topographical differences between experimental factors established using an ANOVA, either reported here or elsewhere, should be interpreted with caution [27]. All of the results that we report are corrected using the Greenhouse-Geisser correction, to account for the potential non-sphericity of EEG data [28]. (Figures show data prior to rescaling.)

## Results

### Behavioural results

**Figure 3:**
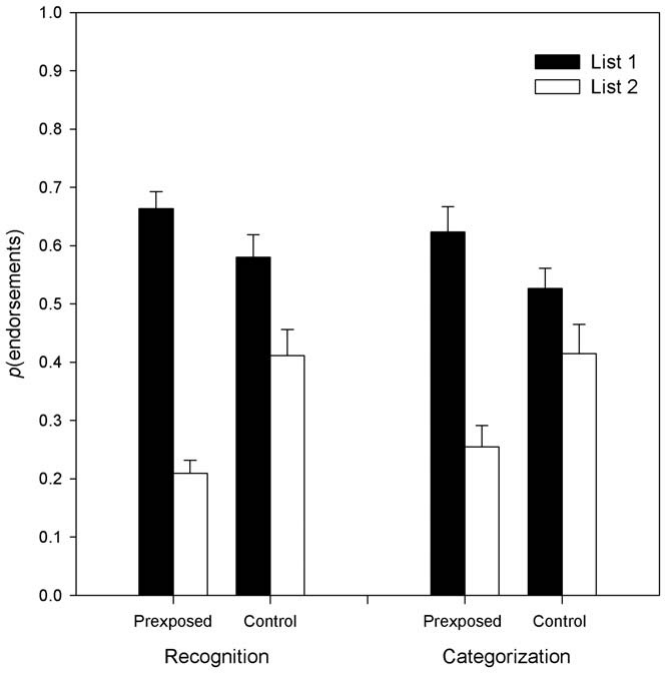
Behavioural Results. Showing the proportions of endorsements for each list for recognition and categorization conditions by exposure. Error bars are +/− SEM.

Responses to items that were identified as category members were treated as endorsements in the categorization condition, and responses to items that were identified as ‘old’ were treated as endorsements in the recognition condition. The mean proportions of endorsements for each List and Condition are shown for each condition in Figure 3. These data were entered into a 2×2×2 mixed model ANOVA with Exposure (Pre-exposed vs. Control) and Instructions (Recognition vs. Categorization) as between-subjects factors and List (1 vs. 2) as a within-subject factor. This revealed a main effect of List (*F*_1, 59_ = 109.08, *MSE*  = .02, *p*<.01, 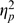
<.65) indicating reliable discrimination between items. A marginal effect of Exposure (*F*_1, 59_ = 2.87, *MSE*  = .02, *p*<.09, 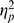
<.05) and a reliable interaction between Exposure and List indicated that discrimination was higher in the Pre-exposed conditions than in the Control conditions (*F*_1, 59_ = 26.41, *MSE*  = .02, *p*<.01, 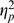
<.31). These results clearly show that discrimination between category members and non-members, and between old and new items is greater following pre-exposure than in control participants.

However, there was no effect of Instructions (*F*_1, 59_ = 0.18, *MSE*  = .02, *p* = .67, 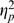
<.01), and no interaction between either Instructions and List (*F*_1, 59_ = 1.82, *MSE*  = .02, *p* = .18, 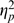 = .03), or between Instructions and Exposure (*F*_1, 59_ = 0.26, *MSE*  = .02, *p* = .61, 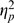
<.01). There was no 3-way interaction (*F*_1, 59_ = 0.08, *MSE*  = .02, *p* = .78, 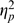
<.01). This aspect of the results is consistent with the view that when items to be recognized are the same as items to be categorized similar patterns of discrimination performance result. This is indicative of the same or similar processes being utilized to make different decisions. That is, categorization is a form of recognition.

To more closely examine discrimination in the four conditions, and to permit a power analysis, we next computed the sensitivity index *d'* by treating endorsements to List 1 items as hits, and endorsements to List 2 items as False Alarms. A comparison of the two pre-exposed groups showed that instructions to recognize or categorize items did not result in a reliable difference in discrimination (*d' = *1.30, vs. 1.20 respectively, *t*_31_ = 0.39, *p = *.70). So that we may be confident that our experiment was sufficiently powerful to detect a possible difference between recognition and categorization we estimated the effect sizes of data from two previous reported comparisons. Both of these made within subject comparisons of these decisions but used different items in each test. The first reported *d′* values of 7.23 and 0.72 for recognition and categorization respectively, from a sample of 4 participants who were acting as controls against an amnesic patient [10]. From the figures that they report we first estimated the pooled standard deviation (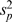 = 0.80) and used this to estimate the effect size (Cohen's d = 7.27). We then calculated the sample size that we would need to find an effect of this magnitude in a between subjects design. The result was 2 participants in each group. Our sample size of 31 easily exceeds this. The second study reported a mean percentage of correct recognition decisions to be 86.0% versus, 64.2% correct categorization decisions, with a sample of 10 and 9 respectively (due to a recording error) [22]. As before we estimated the pooled standard deviation from their reported figures (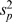 = 11.81). The resulting effect size smaller than the other study but is nonetheless large (Cohen's d = 1.85). The sample size needed to find an effect of this magnitude in a between subjects design is 8 participants in each group. Our sample size also easily exceeds this. The average weighted effect size of both of these studies is Cohen's d = 3.45, and requires a total sample size of just 6 participants. We are therefore confident that had there been a difference in categorization and recognition judgements it would have been detected in our sample of 31 participants. We conclude from this that previous dissociations between categorization and recognition might reflect differences in the test items rather than differences in process.

We also examined whether the control participants were able to discriminate between items by comparing their performance against a chance value of *d' = *0. There was some indication of above chance performance following recognition instructions (*d' = *0.47, *t*_14_ = 3.09, *sd* = .59, *p*<.01), but not following categorization instructions (*d' = *0.32, *t*_14_ = 1.76, *sd* = .71, *p* = .10). This suggests that some abstraction of the category structure can occur during the testing period, in the sense that untrained controls were able to make accurate decisions without any exposure to the study items [29], but this is insufficient to account for the substantially greater number of correct decisions made by the pre-exposed participants.

### ERP Results

**Figure 4:**
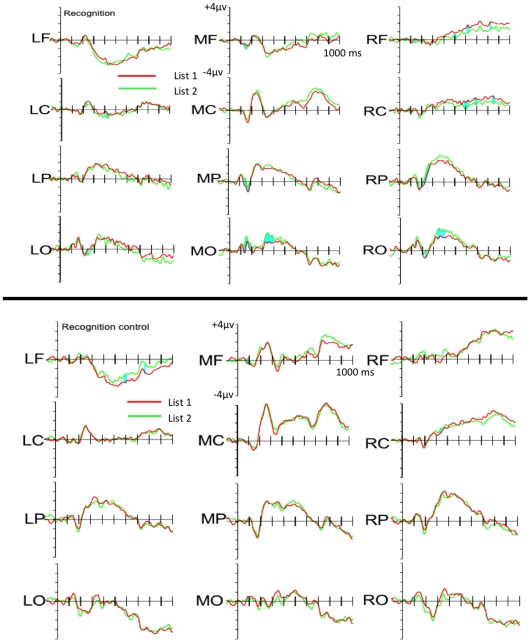
ERPs potentials in the Recognition conditions.

**Figure 5:**
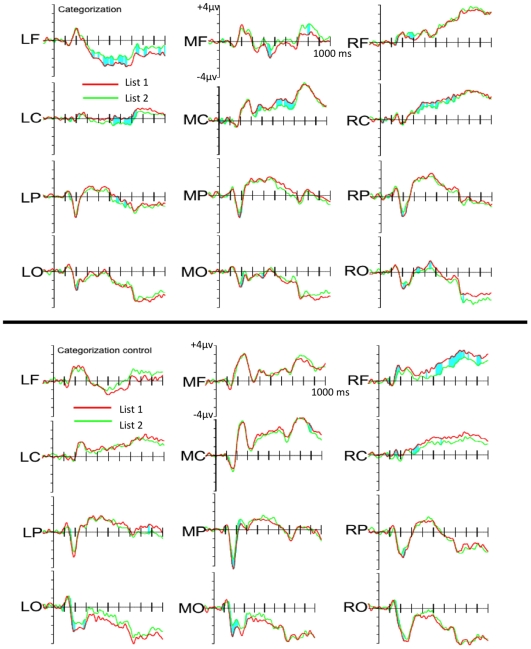
ERPs potentials in the Categorization conditions.

Although the behavioural performance was equivalent across the Recognition and Categorisation groups we were keen to establish whether the ERP data shown in Figures 4 and 5 could distinguish between categorisation and recognition judgements, with and without prior exposure.

#### Early effects (160–200 ms)

Our first comparison was centred on the window most reliably associated with the N1 potential. This had previously distinguished between category members and non-members [17]. In our data, the effect elicited by the items appeared to be more negative in the Categorisation groups than in either of the Recognition groups; more negative in the un-exposed Control groups than in the Pre-exposed groups and more negative for List 1 items than for List 2 items. Furthermore, it appeared that these factors interacted: whilst both the Pre-exposed Categorisation and Recognition groups showed a more negative N1 component for List 1 relative to List 2, of the un-exposed Control groups, only the Categorisation group showed a greater negativity for List 1 relative to List 2.

Our ANOVA revealed main effects of Instruction with the amplitudes in the Categorisation groups being more negative than in the Recognition groups (*F*_1, 56_ = 7.40, *p*<.01), Exposure with the Exposed groups being more negative than the Control groups (*F*_1, 56_ = 4.03, *p*<.05), and List with List 1 eliciting a greater negativity than List 2 (*F*_1, 56_ = 5.93, *p*<.02). There was also a marginally significant interaction between these three factors (*F*_1, 56_ = 3.98, *p*<.06). This resulted from an interaction between List and Instructions in the un-exposed Control groups only (*F*_1, 28_ = 5.04, *p* = .03): there was a relative negativity for List 1 relative to List 2 in the Control Categorisation group (*F*_1, 14_ = 4.84, *p*>.05), but not in the Control Recognition group. By contrast, in the Pre-exposed groups there was no interaction between List and Instructions, there was just a simple main effect of List (*F*_1, 28_ = 4.65, *p* = .04), with List 1 items eliciting a greater negativity relative to List 2 items.

We also observed various interactions between these experimental factors and one or both of the electrode factors. Given the main effects of the experimental factors, we scaled the data and recalculated the ANOVA. An interaction between List and electrode[A-P] survived the normalisation (*F*_2.53, 141.85_ = 3.02, *p* = .04). This resulted from List 1 items being significantly more negative than List 2 items over the parietal (*F*_1, 59_ = 5.47, *p* = .02) and occipital electrode clusters (*F*_1, 59_ = 4.35, *p* = .04), though not over the other electrode clusters. An interaction between List, instruction and electrode[L-R] also survived the normalisation (*F*_1.95, 109.54_ = 3.73, *p*<.03. This resulted from an interaction between List and instruction over the midline electrodes (*F*_1, 58_ = 4.83, *p* = .03), though not over the left or right-hemisphere electrodes. This in turn resulted from a significant negativity for List 1 items relative to List 2 items in the Categorisation groups (F_1, 29_ = 7.34, *p* = .01), though not for the Recognition groups.

#### Mid-latency effects (320–480 ms)

There were no main effects of any of the experimental factors within this time window. However, there were two interactions between the experimental and electrode factors: prior exposure interacted significantly with electrode[L-R] (*F*_1.72, 96.50_ = 3.87, *p* = .03). This was not driven by any one cluster significantly, though List 1 items elicited a marginally more negative amplitude over the midline electrodes in the Exposed groups relative to the Control groups (*F*_1, 56_ = 3.43, *p* = .06). We also observed a marginally significant interaction between List, electrode[L-R] and electrode[A-P] (*F*_3.53, 197.72_ = 2.45, *p* = .05). This was because List 1 items elicited a greater positivity than List 2 items over the right-hemisphere central cluster (*F*_1, 56_ = 12.73, *p*<.01), but not over any other clusters.

#### Late-latency effects (480–800 ms)

There were no main effects or interactions between any of the experimental factors. There were two significant interactions between experimental and electrode factors: Instruction, Exposure and electrode[L-R] interacted significantly (*F*_1.55, 86.83_ = 3.67, *p* = .04). There was no clear effect driving this interaction, though the closest to reaching significance was an interaction between Exposure and electrode[L-R] in the Categorisation groups (*F*_1.37, 38.33_ = 3.44, *p* = .06), which had not been present in the Recognition groups. This marginal effect was, in turn, driven by relatively more negative amplitudes for the Exposed, relative to the un-exposed Control group, over the left-hemisphere clusters (*F*_1, 28_ = 3.96, *p* = .05). We also noted a significant interaction between List, electrode[L-R] and electrode[A-P] (*F*_3.94, 220.87_ = 5.62, *p*<.01). This was the result of a significant negativity over the left frontal cluster (*F*_1, 58_ = 5.45, *p* = .02), and a significant positivity over the right frontal (*F*_1, 58_ = 7.24, *p*<.01) and central (*F*_1, 58_ = 7.31, *p*<.01) clusters, for List 1 items relative to List 2 items.

## Discussion

The aim of this experiment was to examine the ERPs of categorization and recognition in the well-known prototype-distortion task [7]. Previous studies have used either amnesic patients [5] or *f*MRI [6] to dissociate categorization from recognition. To our knowledge this is the first to use ERP to do so, although one previous study has used a less well known paradigm to examine the same processes [17]. These previous studies have compared recognition judgements made to one set of items to categorization judgements made to a different set of items. The resulting differences are frequently cited as evidence that prototype knowledge is used to make categorization decisions using a separate process than episodic memory of the study items that is used to make recognition judgements. This particular experimental preparation makes the interpretation of dissociations involving amnesic patients or *f*MRI difficult because they may be due, as is claimed, to the processes involved, or to differences in the stimuli themselves. In this experiment we sought to resolve this problem by asking participants to make recognition and categorization judgements to the same set of stimuli. The participants were allocated to four groups were presented with the same category members and non-members (termed ‘List 1’ and ‘List 2’ items, respectively). A key finding was that participants were equally able to distinguish List 1 from List 2 items, regardless of whether they had been asked to attempt to categorise or recognise them. This is in contrast to previous studies that have compared these decisions to different items [10], [22]. We did not replicate these effects despite sufficient experimental power to do and so conclude that the dissociations reported previously could be due to differences in the stimuli rather than difference in the processes used to judge them. Nonetheless, previous studies have reported activity in different brain regions dependent on the decision that participants are asked to make, and although we observed no differences in the decisions that the participants made this does not preclude differences in the neural processes used to make those decisions. Indeed the ERP data from our experiment demonstrated that the early visual evoked response that distinguished category members and non-members was modulated by whether participants had been asked to categorise or recognise them, and, although not to the same extent, by whether participants had previously been exposed to those items.

### 

#### Influence of instruction and prior exposure on early visual processing of items

In the window usually associated with the N1 potential, List 1 elicited a significantly more negative potential than did List 2 items. This was most prominent over the parietal and occipital electrodes. However, unlike previous research [17], we found that the N1 amplitude was modulated by more than just category membership. Whilst the early effect of List was not affected by judgement for both the Pre-exposed Recognition and Categorisation groups, the effect was only present for the Control Categorisation group. The Control Recognition group showed no such early differentiation between List 1 and List 2 items. This result overlapped with another result: taken together the Categorisation groups showed an increased negativity for List 1 relative to List 2 items over the midline electrodes, whereas the Recognition groups did not, presumably because whilst both Categorisation groups showed the effect, only the Pre-exposed Recognition group did. Effects driven primarily by changes to the N1 amplitude are typically ascribed to early visual processing in the extrastriate cortex [18]. Indeed, recent *f*MRI studies have linked early visual processing with prototype abstraction/application [6], [12]. Interestingly, we observed these effects for both Categorisation groups, regardless of whether or not they had previously been exposed to the category members. Possibly, when the participants' task is to categorise items, prototype abstraction/application can occur within the test phase. This would explain why even the un-exposed Control Categorisation group showed the N1 amplitude differentiation for List 1 and List 2 items. By contrast, this might not be the case when the participants' task is to attempt to recognise the items. In this case, participants only engage in prototype abstraction/application when they have already had some experience of category members. This account makes intuitive sense: when the task is to categorise abstract items based upon arbitrary but consistent perceptual characteristics, the participant will pay close attention to those consistent characteristics that distinguish category members from non-members – this is the case even these have not been encountered previously. By contrast, when the task is to ‘recognise’ abstract items, the participant might only proceed with prototype abstraction/application when it becomes apparent that those membership-defining characteristics discriminate between the items that were studied earlier form those that were not. That is, when attempting to recognise the items, participants will only engage in this process when they have already been exposed to the category members. The Control group might simply not realise the prototype abstraction/application is beneficial for their ‘recognition’ task.

When one observes an effect that can occur in both Pre-exposed Categorisation and Recognition groups, it might be tempting to take this as evidence that both tasks tap some common recognition-like mechanism. However, in the case of this early N1 effect, this would not explain why the Control Categorisation group also show the effect: they cannot be recognising items that they have not seen before. By contrast, both the Pre-exposed Categorisation and Recognition groups could be employing a categorisation-like strategy. With our stimuli and procedure such a strategy would be successful for both of the Pre-exposed groups, but only for the Control Categorisation group. The Control Recognition group, in contrast to the Pre-exposed Recognition group, would have no category for “old” items and therefore would not be able to use this strategy. Interestingly, this is precisely the pattern of N1-like effects that we observed.

#### Influence of instruction and prior exposure on later potentials

The effects elicited by List 1 and List 2 items also differed later in the epoch: there was a greater positivity for List 1 relative to List 2 items over the right-hemisphere central electrode cluster, between 320 and 480 ms. Again, later in the epoch, between 480 and 800 ms, List 1 items elicited a negativity over the left-hemisphere and positivity over the right-hemisphere, relative to List 2 items. Whilst the time window corresponds to the differences reported in previous papers [17], the effects themselves are quite different in terms of distribution and amplitude in our data. Unlike the early N1-like effects, the later effects were primarily driven by differences between List 1 and List 2 items, regardless of which judgement participants performed and their prior exposure to category members. At least with regard to these later effects, they appear to reflect some process common to categorisation and recognition, a conclusion perhaps supported by the similarity in behavioural performance between the two judgement types.

### Conclusions

Participants were equally good at distinguishing category members from non-members, regardless of whether they were performing a categorisation or recognition judgement. This result contrasts shapely with previous studies that have reported differences between categorization and recognition judgements. However, the ERPs suggested that participants' early visual potentials (160–200 ms), often associated with prototype abstraction/application, distinguished category members from non-members in both the Pre-exposed Recognition and Categorisation groups. By contrast, in the un-exposed Control groups, only the participants explicitly asked to categorise the items showed this early visual differentiation of members and non-members – the un-exposed Control Recognition group did not. One possible interpretation of these data is that prototype abstraction/application occurs on both categorisation and recognition tasks, but only when participants have actually been pre-exposed to category members. If they have not been pre-exposed then prototype abstraction/application will only occur in a categorisation task. The data suggest that both categorization and recognition in prototype distortion tasks appear to rely on the same underlying process.
